# Weight cycling induces innate immune memory in adipose tissue macrophages

**DOI:** 10.3389/fimmu.2022.984859

**Published:** 2023-01-11

**Authors:** Heather L. Caslin, Matthew A. Cottam, Jacqueline M. Piñon, Likem Y. Boney, Alyssa H. Hasty

**Affiliations:** ^1^ Department of Molecular Physiology and Biophysics, Vanderbilt University, Nashville, TN, United States; ^2^ Vanderbilt Center for Stem Cell Biology, Vanderbilt University, Nashville, TN, United States; ^3^ Veterans Affairs, Tennessee Valley Healthcare System, Nashville, TN, United States

**Keywords:** obesity, weight loss, weight cycling, innate immune memory, trained innate immunity, adipose tissue macrophages

## Abstract

**Introduction:**

Weight loss improves obesity-associated diabetes risk. However, most individuals regain weight, which worsens the risk of developing diabetes and cardiovascular disease. We previously reported that male mice retain obesity-associated immunological changes even after weight loss, suggesting that immune cells may remember the state of obesity. Therefore, we hypothesized that cycles of weight gain and loss, otherwise known as weight cycling, can induce innate memory in adipose macrophages.

**Methods:**

Bone marrow derived macrophages were primed with palmitic acid or adipose tissue conditioned media in a culture model of innate immune memory. Mice also put on low fat or high fat diets over 14-27 weeks to induce weight gain, weight loss, and weight cycling.

**Results:**

Priming cells with palmitic acid or adipose tissue conditioned media from obese mice increased maximal glycolysis and oxidative phosphorylation and increased LPS-induced TNFα and IL-6 production. Palmitic acid effects were dependent on TLR4 and impaired by methyltransferase inhibition and AMPK activation. While weight loss improved glucose tolerance in mice, adipose macrophages were primed for greater activation to subsequent stimulation by LPS *ex vivo* as measured by cytokine production. In the model of weight cycling, adipose macrophages had elevated metabolism and secreted higher levels of basal TNFα, suggesting that weight loss can also prime macrophages for heighted activation to weight regain.

**Discussion:**

Together, these data suggest that weight loss following obesity can prime adipose macrophages for enhanced inflammation upon weight regain. This innate immune memory response may contribute to worsened glucose tolerance following weight cycling.

## Introduction

Weight loss is effective for improving blood glucose, blood pressure, and blood lipids (1–3). However, weight loss is hard to accomplish and even harder to maintain, and most individuals regain weight within a few years (4–7). We refer to the repeated process of gaining and losing weight as weight cycling, which can occur throughout one’s lifetime. Unfortunately, weight cycling further increases the risk for developing type 2 diabetes, cardiovascular disease, and hypertension (8–11), and has a stronger relationship with all-cause mortality than even stable long-term obesity (12). However, the mechanism by which weight cycling worsens disease risk remains unknown.

Obesity is a state of chronic, low-grade, systemic inflammation, and adipose immune cells contribute to obesity-associated disease. Upon weight gain, inflammatory cells expand and infiltrate into the adipose tissue and release inflammatory cytokines such as IL-1β, TNFα, and IL-6 (13–18). These cytokines directly promote adipocyte lipolysis and impair insulin signaling, contributing to the development of diabetes (19). We previously published our work demonstrating that male C57BL/6J that undergo weight cycling have worsened glucose tolerance compared with their obese counterparts, and this is correlated with an increase in memory T cells in their adipose tissue (20). We have also reported that while weight loss normalizes glucose tolerance, it does not restore obesity-associated immunological changes such as T cell exhaustion or macrophage lipid handling (21). These data suggest that adipose immune cells may “remember” the state of obesity.

The induction of immune memory has long been known to be a key feature of T cells and B cells. However, recent studies have also revealed a memory response in innate immune cells, in which stimuli prime innate immune cells to augment subsequent activation to a second stimulus. This response, coined “innate immune memory” or “trained innate immunity”, was initially observed with β-glucan and the *Mycobacterium tuberculosis* vaccine (BCG), but has also been seen with cytokines, hormones, and oxidized low density lipoprotein (22–28). Functionally, innate immune memory is associated with elevated glycolytic metabolism and inflammatory function. Mechanistically, this memory response is controlled by epigenetic modifications that hold open regions of chromatin at glycolytic and inflammatory genes (28). We hypothesized that weight cycling induces innate immune memory in adipose tissue macrophages and could contribute to worsened disease risk.

In the present study, we show that previous exposure to palmitic acid or adipose conditioned media from obese mice *in vitro* and weight loss *in vivo* increase macrophage metabolism and inflammatory cytokine production. This immunological memory may influence weight maintenance or the metabolic consequences of further weight gain.

## Methods

### Animals

Male and female C57BL/6J mice were either purchased from Jackson Labs or bred within our group. TLR4 KO mice on a C57BL/6 background were provided by Dr. Brad Grueter at Vanderbilt Universtiy (29). All procedures were approved and carried out with approval from and in compliance with the Vanderbilt University Institutional Animal Care and Use Committee. Vanderbilt University is accredited by the Association for Assessment and Accreditation of Laboratory Animal Care International.

### Weight cycling

**Figure 1:**
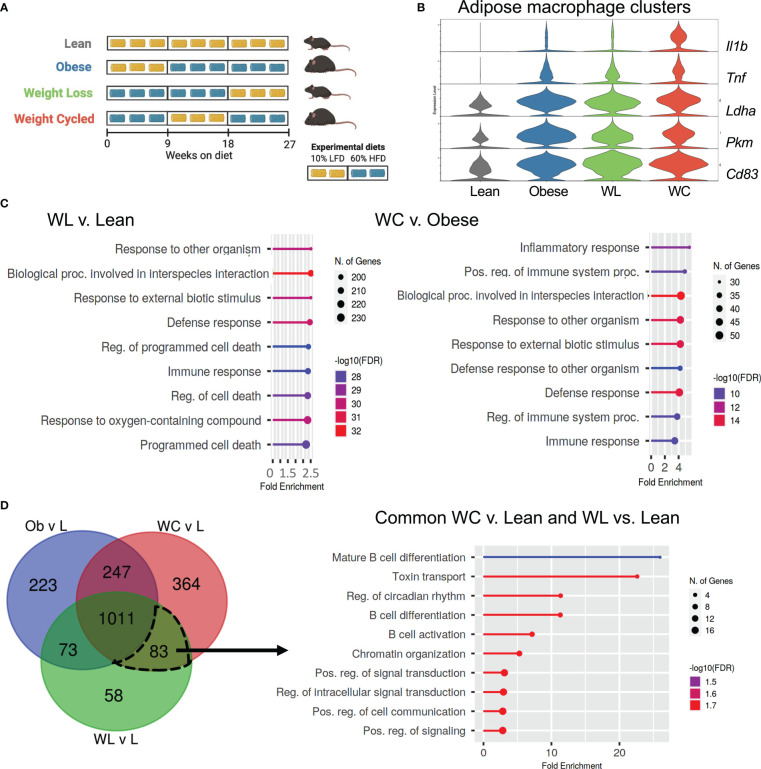
Transcriptional changes in adipose macrophages from weight loss (WL) and weight cycled (WC) mice suggest innate immune memory. **(A)** Weight cycling schematic. **(B)** Violin plot of inflammatory (*Tnf, Il1b, Cd83*) and metabolic genes (*Ldha, Pkm*) from lean, obese, weight loss (WL), and weight cycled (WC) macrophage clusters. **(C)** Pathway analysis of differentially expressed genes from WC vs. obese or WL vs. lean macrophage clusters. **(D)** Venn diagram of differentially expressed genes of obese, WL, and WC adipose macrophage clusters compared with lean and pathway analysis of the genes differentially expressed in both WL and WC groups.

For weight loss and weight cycling experiments, male C57BL/6J mice were purchased from Jackson Labs (#000664) at 7 weeks of age. At 8 or 9 weeks of age, mice were placed on 9-week cycles of high fat diet (60% fat, Research Diets #D12492, 5.21 kcal/g food) or low fat diet (10% fat, Research Diets #D12450B, 3.82 kcal/g) for a total of 27 weeks as published (20, 21) and as visualized in **Figure 1A**. Access to food and water was provided *ad libitum*. Body weight and food intake were recorded weekly.

### Single cell analysis

To assess adipose macrophage populations for innate immune memory by single cell-sequencing, we used a previously published single cell sequencing dataset from our laboratory (GEO accession number GSE182233) (21). Differential expression was performed in R version 4.1 with Seurat V4 (30) using Wilcoxon Ranked Sum tests. GO Biological Processes pathway analysis was conducted on differential expression with ShinyGO 0.76 (31) using FDR cutoff of 0.05 (last date of access 6/15/2022) and overlapping gene expression was determined and graphed using http://bioinformatics.psb.ugent.be/webtools/Venn/.

### Bone marrow derived macrophages and adipose tissue conditioned media

For innate immune memory culture models, male and female mice on chow diets were euthanized between 8-15 weeks of age and bone marrow was extracted from the femurs. Bone marrow-derived macrophages (BMDM) were differentiated over 5-7 days in DMEM (Gibco, 11960044 or 11885092), 10% FBS, 1% Penicillin/streptomycin, 10 mM HEPES, and 10% L929 conditioned media (or 10 ng/mL M-CSF; Shenandoah Biotech #200-08) in T75 flasks.

For adipose tissue conditioned media, male mice were fed 10% low fat diet or 60% high fat diet as above for 5-9 weeks were euthanized and epididymal adipose fat pads were collected and minced. Small pieces of adipose tissue were cultured in DMEM with FBS, Pen/strep, and HEPES as above for 48 hours at ~50 mg/mL. Media was filtered through 50 μm filters to remove adipocytes, centrifuged to remove red blood cells and immune cells, and stored at -20°C for further experiments.

### *In vitro* innate memory model

For *in vitro* innate immune memory experiments, we adopted a cell culture model by Kleinnijenhuis, Quintin, and colleagues (23,24). Briefly, BMDM were treated for 24 hours with 0.4 mM palmitic acid (MP Biomedicals #57-10-3) or 100% adipose tissue conditioned media. Palmitic acid was suspended in DMSO or conjugated to BSA. Media was washed out for 6 days and cells were stripped, re-plated at 375 cells/μL, and activated with 100 ng/mL LPS (Sigma #L4391), 1μg/mL lipotechoic acid (*In vivo*Gen #tlrl-pstla), 1 μg/mL poly(I:C) (Tocris #4287), or 5 μg/mL beta-glucan (Millipore #346210) for 24 hours. Seahorse metabolic analysis was conducted at the end of the washout phase and cell culture media was collected at the end of 24-hour activation. For mechanistic experiments, BMDM were treated with 0.5 mM 5’-methylthioadenosine (MTA; Sigma #D5011) or 10 μM metformin (Tocris #2864) during the initial activation phase with palmitic acid.

### Seahorse metabolic analysis

To measure cell metabolism, a Seahorse XFe96 (Agilent) was used to measure the extracellular acidification rate (ECAR) and oxygen consumption rate (OCR) as surrogates for glycolysis and oxidative phosphorylation, respectively. Cells were plated at 50-75,000/well in 200 μL of standard growth media for 2-24 hours and then switched to minimal DMEM containing 2 mM L-glutamine for the assay. A modified version of the Mito Stress Test (Agilent #103015-100) was then performed. Briefly, glucose (final concentration 10 mM) was injected to the wells to measure glucose-stimulated glycolysis. Pyruvate (final concentration 1 mM) was then injected with oligomycin (final concentration 1.5 μM) to begin the standard Mito Stress Test. Last, FCCP (final concentration 2 μM) and rotenone/antimycin A (final concentration 0.75 μM) were injected. To normalize for any differences in cell number due to treatment, cells were lysed in RIPA buffer and we performed a Pierce BCA assay (Thermofisher #23225) according to the manufacturers’ protocol. OCR and ECAR were normalized to μg protein.

### ELISA

To measure cytokine production, cell culture media was collected for ELISA. IL-6 and TNF murine ELISA kits were purchased from Biolegend (#431304, #430904). Assays were performed in duplicate when possible, according to the manufacturers’ protocols. To normalize for any differences in cell number due to treatment, cells were lysed in RIPA buffer and a Pierce BCA assay (Thermofisher #23225) performed according to the manufacturers’ protocol. Cytokine concentrations were normalized relative to control samples from each experiment.

### Body composition

To assess body composition, mouse body fat and fat free mass (FFM) were measured by nuclear magnetic resonance whole body composition analysis (Bruker Minispec).

### Glucose tolerance testing

To assess glucose tolerance, mice were fasted for 5 hours and anesthetized with isoflurane for a tail snip to access blood supply. After a 1-hour recovery, basal blood glucose levels were measured using a hand-held glucometer (Bayer Contour Next EZ meter). After an intraperitoneal injection of 1.5 g dextrose/kg lean mass, blood glucose was sampled at 15, 30, 45, 60, 90, and 120 minutes.

### Tissue immune cell isolation

To isolate adipose macrophages, the adipose stromal vascular fraction was collected as previously described (32). Briefly, mice were euthanized by isoflurane overdose and cervical dislocation and perfused with 20 ml PBS through the left ventricle. Epididymal (unless otherwise stated) or subcutaneous adipose depots were collected, minced, and digested in 6 ml of 2-mg/mL type II collagenase (Sigma # C6885 or Worthington #LS004177) for 30 min at 37°C. Digested tissue was then vortexed, filtered through 100 μm filters with cold PBS, lysed with ACK buffer, and filtered through a 35 μm filter. The stromal vascular fraction was plated at 200,000 cells/well for 2-4 hours to allow for adherence for Seahorse and ELISA experiments or stained for flow cytometry.

To isolate blood monocytes, whole blood was lysed in water and 500,000 cells/well were plated for 2 hours for adherence and used for cytokine production.

To isolate peritoneal macrophages, peritoneal lavage was performed. Cold PBS (5 mL) with 5mM EDTA was injected into the abdominal cavity, the abdomen was massaged for 2 min, and lavage fluid was collected from a small incision by transfer pipette. 200,000 cells/well were plated for adherence for 2 hours for Seahorse metabolic analysis and cytokine production.

To isolate liver macrophages, livers were extracted, minced, and digested as above. The liver suspensions were then plunged through 100 μm filters with cold PBS and lysed with ACK buffer. The cell pellet was resuspended in 33% Percoll and overlayed on top of 66% Percoll. The Percoll gradient was centrifuged at 600 x g for 15 min with the break set to zero. The two middle layers of the Percoll gradient were collected in HBSS and centrifuged at 500 x g for 5 min. 200,000 cells/well were plated for 2 hours for adherence and used for Seahorse metabolic analysis and cytokine production.

### Flow cytometry

To assess cell frequency and intracellular cytokine production, stromal vascular cells were prepared for flow cytometry. FcBlock was added for 10 min prior to fluorescent antibody staining for surface proteins for 30 min at 4°C using the following antibodies:

BV510 anti-mouse CD45 (Biolegend 103137), FITC anti-mouse CD11b (eBioscience 11-0112-86), PerCP-Cy5.5 anti-mouse CD64 (Biolegend 139308), PE-Cy7 anti-mouse CD9 (Biolegend 124815), APC-Cy7 anti-mouse F480 (Biolegend 123118). eFlour 450 fixable viability dye (Invitrogen/eBioscience 50-112-8817 was used to determine viability). For intracellular cytokines, cells were fixed and permeabilized according to the Thermofisher two-step protocol: for intracellular (cytoplasmic) proteins (Foxp3/transcription factor staining buffer set, eBioscience 00-5523-00). Cells were then stained with fluorescent antibodies for intracellular proteins for 30 min at 4°C using the following antibodies: PE anti-mouse TNF (Biolegend 506306) and APC anti-mouse IL-6 (Biolegend 503810). Data was acquired on a MACSQuant10 (Miltenyi) and analyzed on FlowJo.

### Statistical analyses

Statistical analyses were performed using GraphPad Prism. Student’s *t*-tests were run for comparisons between two groups, one-way analysis of variances (ANOVA)s were used for comparisons between more than two groups, and two-way ANOVAs were conducted for >2 groups over >2 timepoints. For significant main effects, *post-hoc* pairwise comparisons using Tukey or Sidak corrections were used to determine statistical differences. All data are presented as mean ± standard error (SEM). A *p*-value (or adjusted *p* -value) of <0.05 was used to determine significance.

## Results

### Transcriptional induction of innate immune memory in adipose macrophages from weight loss and weight cycled mice

To determine if weight cycling can induce innate immune memory, we first utilized a recently published single cell dataset from our lab (21). Briefly, mice had been placed on low fat or high fat diets for 27 weeks total (**Figure 1A**). At the end of 27 weeks, obese and weight cycled mice were matched for body weight, and obese, weight loss, and weight cycled mice were matched for total time on high fat diet. Single-cell sequencing was completed on CD45^+^ adipose tissue immune cells and analyses across many different cell populations were reported (21). To extend upon those findings, genes from the Trained Immunity DataBase (33) were plotted across our diet groups in the macrophage clusters (**Figure 1B**). We performed differential expression analysis and found inflammatory genes (*Il1b* and *Tnf*), glycolytic genes (*Pkm* and *Ldha*), and the activation marker *Cd83* were more highly expressed in adipose macrophages from weight loss compared to lean mice (adjusted p<0.05 by differential expression, **Supplemental Table 1**). Additionally, *Il1b* and *Cd83* were more highly expressed in the adipose macrophages from weight cycled vs. obese mice (adjusted p<0.05 by differential expression, **Supplemental Table 2**). Next, we performed pathway analysis on all significant differentially expressed genes to determine which pathways were differently regulated (**Figure 1C**). In weight loss compared with lean as well as weight cycled compared with obese, pathways related to immune regulation, inflammation, defense, and responses to biotic, external stimuli, and interspecies interaction were changed. These pathways have also been shown to change following the induction of innate immune memory by β-glucan or BCG (34, 35). To further determine the pathways changed in both weight loss and weight cycled groups, we used a Venn diagram to compare the differentially expressed genes in all groups when compared to the lean group (**Figure 1D**). Of the 83 genes that were significantly different in both the weight loss and weight cycled groups, common pathways were related to chromatin organization, signal transduction, and cell communication. These cellular processes are all generally considered to be characteristic of innate immune memory. Interestingly, we also found pathways associated with differentiation and activation in B cells and toxin transport. Together, these data supported our hypothesis that weight loss and weight cycling may induce innate immune memory in adipose macrophages.

### Transcriptional induction of innate immune memory in monocytes and dendritic cells

Using the dataset from **Figure 1**, we also completed the same analyses on the monocyte and dendritic cell (DC) clusters (**Figures S1A, B**). *Il1b* was significantly increased in classical monocytes from weight cycled vs. obese mice (**Supplemental Tables 3, 4**), and both *Il1b* and *Tnf* were more highly expressed in the cDC2 and monocyte derived DC populations from weight cycled vs. obese (adjusted *p*-value <0.05, **Supplemental Tables 5, 6**). In the classical monocytes, pathways related to immune regulation, defense, responses to biotic or external stimuli were changed similar to adipose macrophages in **Figure 1B** and to the response following β-glucan or BCG (34, 35) (**Figure S1C**). Notably, cytokine response genes and metabolic processes were also changed. In the cDC2 and monocyte derived DC clusters, many of the pathways with the greatest change were related to translation, protein biosynthesis/metabolism, and cytokine response. In the clusters from weight cycled vs. obese mice, differences were observed in pathways related to innate receptor signaling and inflammation. These data further support the hypothesis that weight cycling may induce innate immune memory in innate myeloid cells of the adipose tissue.

We also looked at NK cell populations, which display evidence of training in other settings (36); however, only *Malat1* and *Zfp36l2* were significantly different between weight cycling and obese conditions in our NK cells (**Supplemental Tables 7, 8**). There was no detectable *Il1b* or *Tnf* (or even *Csf1* or *Ifna1*), and pathway analysis did not show changes in anything related to pathogen defense or inflammatory, therefore it does not appear that weight cycling affects innate immune memory in NK cells.

### Palmitic acid induces innate immune memory responses in bone marrow derived macrophages

**Figure 2:**
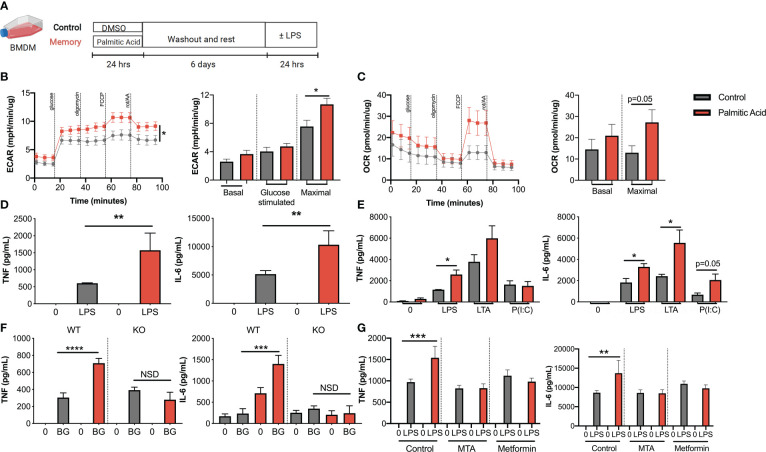
Palmitic acid increases maximal cell metabolism and inflammation in macrophages in culture. **(A)** Schematic of innate immune memory *in vitro* models using bone marrow derived macrophages and 0.4 mM palmitic acid or control (DMSO). **(B)** Extracellular acidification rate (ECAR) and **(C)** oxygen consumption rate (OCR) during modified mitochondrial stress test by Seahorse metabolic analyzer after 6-day washout **(D)** 100 ng/mL LPS- induced TNF and IL-6 production by ELISA and normalized by protein concentration. **(E)** 100 ng/mL LPS-, 1 μg/mL LTA- or 1μg/mL P(I:C)- induced TNF and IL-6 production by ELISA and normalized by protein concentration. **(F)** 100 ng/mL LPS-induced TNF and IL-6 production by ELISA in WT or TLR4-KO mice and normalized by protein concentration. **(G)** 0.5 mM 5’-methylthioadenosine (MTA) or 10 μM metformin were added during the initial 24-hour treatment with palmitic acid. TNF and IL-6 production was measured by ELISA after 100 ng/mL LPS activation. Data are means ± SEM of 3-7 populations, representative of 2-3 independent experiments. *p < 0.05, **p < 0.01, *** *p <*0.001, ****p <.0001, NSD= no significant difference.

We next adopted a previously published cell culture model of innate immune memory with a 24-hour prime, 6-day washout, and 24-hour secondary activation (**Figure 2A**) (23, 24). Palmitic acid was chosen as our initial stimulus because it is the most common saturated fatty acid in the human body, is elevated in individuals with obesity due to lipolysis, and it activates TLR4, a receptor that can promote innate immune memory (37). Additionally, palmitate has been shown to elicit an obese-like adipose macrophage phenotype in bone marrow derived macrophages (BMDM) and priming in other models (38–40). Following 24-hour activation and a 6-day washout, palmitic acid priming increased maximal extracellular acidification rate (ECAR), a proxy for glycolysis, and modestly increased maximal oxygen consumption rate (OCR), a proxy for oxidative phosphorylation, although this measure was not significant (**Figures 2B, C**). Moreover, following 24-hour LPS activation, palmitic acid priming increased LPS-induced TNFα and IL-6 secretion when compared to the control (**Figure 2D**). These results were observed whether palmitic acid was suspended in DMSO (shown) or BSA (data not shown). Cytokine production was also generally increased with palmitic acid priming in response to additional secondary stimuli such as lipoteichoic acid, a TLR2 agonist (**Figure 2E**), as well as β-glucan, a dectin-1 agonist (**Figure 2F**), although there were cytokine specific effects. We did not detect an increase in response to poly(I:C), a TLR3 agonist, suggesting that the priming effect influences a variety of, but not all, stimuli (**Figure 2E**).

To determine potential mechanisms of action, we first tested if palmitic acid requires the innate receptor TLR4. There was no significant difference between palmitic acid primed or control BMDM activated with β-glucan in TLR4 KO BMDM (**Figure 2F**), suggesting that this response is dependent on palmitic acid activation of TLR4. As expected, there was no LPS activation in the TLR4 KO (data not shown). Additionally, we tested the role of epigenetic and metabolic changes within our system. 5’- methylthioadenosine (MTA), which broadly inhibits methyltransferase activity, or metformin, which activates AMPK, inhibits mTOR activity, and suppresses glycolysis in immune cells, were added during the initial 24-hour treatment with palmitic acid. Following subsequent LPS activation, there was no significant difference between palmitic acid primed or control BMDMs (**Figure 2G**). Together, these data suggest that palmitic acid increases metabolic potential and cytokine production to a secondary stimuli, which is consistent with other innate immune memory stimuli (23, 24). Moreover, this form of innate memory is driven by TLR4 activation as well as metabolic and epigenetic changes.

Becker’s group has previously published that 24-hour treatment of BMDMs with palmitate induces metabolically activated macrophages (MMe) with elevated gene and protein expression of Plin2, Abca1, and Cd36 that is consistent with adipose macrophages from obese mice (38, 39). To confirm that our model of enhanced LPS-responsiveness is distinct from MMe activation, we compared 24-hour treatment with priming as above. Mme activation did not increase LPS-induced IL-6 or TNFα (**Figure S2**), suggesting that our innate immune memory model, and potentially weight cycling, is immunologically distinct from models of simple obesity.

### Adipose tissue conditioned media increases cell metabolism and inflammation in bone marrow derived macrophages

**Figure 3:**
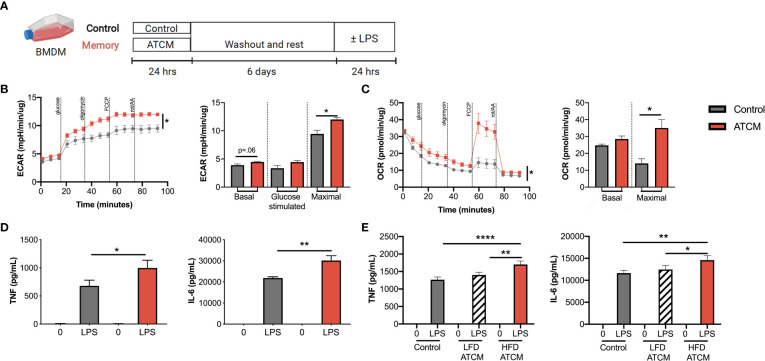
Adipose conditioned media increases cell metabolism and inflammation in macrophages and DCs in culture. **(A)** Schematic of innate immune memory *in vitro* models using bone marrow derived macrophages and adipose tissue conditioned media (ATCM) or control media. **(B)** Extracellular acidification rate (ECAR) and **(C)** oxygen consumption rate (OCR) during modified mitochondrial stress test by Seahorse metabolic analyzer. **(D)** TNF and IL-6 production induced by 100 ng/mL LPS as measured by ELISA. Data are normalized by protein concentration. **(E)** BMDM were treated with ATCM from mice on a high fat diet as in B-D or low fat diet and compared against control media. TNF and IL-6 production were measured by ELISA after 100 ng/mL LPS activation. Data are means ± SEM of 3-4 populations, representative of 2-3 independent experiments. *p <0.05, **p <.01, ****p < 0.001.

Innate immune memory has been shown in response to pattern recognition receptors like TLRs, but also in response to aldosterone, catecholamines, glucose, and cytokines (27, 41–44). With obesity, there is altered levels of catecholamines, glucose, and cytokines such as IFNγ, but also hormones like leptin and insulin. Thus, we extended our above model to a more physiological model for weight cycling by priming cells with adipose tissue conditioned media (ATCM) from obese mice (**Figure 3A**). Priming BMDM with ATCM from obese mice significantly increased maximal glycolysis and oxidative phosphorylation compared with media alone (**Figures 3B, C**). Priming with ATCM also increased LPS-induced TNFα and IL-6 secretion (**Figure 3D**). Importantly, ATCM from lean mice matched for age and time on diet did not induce innate immune memory (**Figure 3E**), suggesting that the priming signal is present or elevated in the obese adipose tissue environment specifically. These data further confirm that repeated exposure to an obese adipose tissue environment increases metabolic potential and cytokine production, which is again consistent with other innate immune memory stimuli.

### Weight loss mimics innate immune memory in adipose macrophages

**Figure 4:**
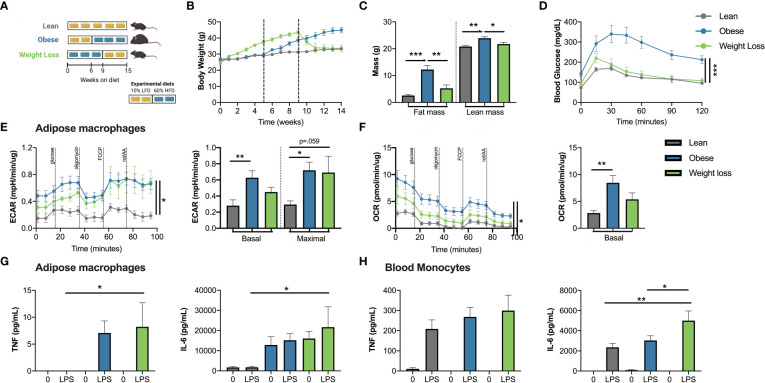
Weight loss doesn’t normalize the inflammatory response in adipose macrophages. **(A)** Weight loss *in vivo* schematic. **(B)** Body mass over time measured weekly with diet switch indicated by dashed lines. **(C)** Lean and fat mass measured by nuclear magnetic resonance. **(D)** Blood glucose during an intraperitoneal glucose tolerance test (1.5 g dextrose/kg lean mass) at 14 weeks. **(E)** Extracellular acidification rate (ECAR) and **(F)** oxygen consumption rate (OCR) of epididymal adipose macrophages selected by 2 hour adherence during modified mitochondrial stress test by Seahorse metabolic analyzer. **(G)** TNF and IL-6 production induced by 100 ng/mL LPS as measured by ELISA in epididymal adipose macrophages selected by 2-hour adherence and normalized to protein concentration. **(H)** TNF and IL-6 production induced by 100 ng/mL LPS as measured by ELISA in monocytes selected by 2-hour adherence and normalized to protein concentration. Data are means ± SEM of 6-8 mice, representative of 2 independent experiments. *p <0.05, **p < 0.01, ***p <0.001.

We next tested if weight loss could replicate the effects observed when priming cells with palmitate and ATCM above. Mice were placed on low fat diet or high fat diet as in **Figure 4A**. After 9 weeks of high fat diet, we observed the predicted increase in body mass, fat mass, and lean mass in obese mice (**Figures 4B, C**). After switching to a low fat diet for 5 weeks, our weight loss mice had similar body weight and body composition to mice fed low fat diet for the entire 14 weeks (**Figures 4B, C**). During an intraperitoneal glucose tolerance test, obese mice had higher blood glucose and slower glucose disposal at all timepoints compared to lean mice; however, glucose tolerance was not significantly different in weight loss mice compared to lean mice (**Figure 4D**). Obesity increased glycolysis and oxidative phosphorylation in adipose macrophages as expected (45, 46) (**Figures 4E, F**). Maximal glycolysis trended toward remaining elevated in adipose macrophages from weight loss mice, although this did not reach statistical significance. Strikingly, adipose macrophages from weight loss mice increased LPS-induced TNFα and IL-6 production to a degree similar to cells from obese mice, while adipose macrophages from lean controls demonstrated almost no response (**Figure 4G**). Weight loss also significantly increased LPS-induced IL-6 production in blood monocytes (**Figure 4H**). Interestingly, these results were tissue specific, as peritoneal macrophages and liver macrophages had similar metabolism and cytokine production across groups (**Figure S3**). Moreover, in an extended model of weight loss (18 weeks), adipose macrophages still trended towards increased metabolic and inflammatory parameters, although only LPS-induced TNFα production was statistically elevated compared to the lean group (**Figure S4**). Together, these data suggest that while weight loss improves glucose tolerance, it increases inflammatory cytokine production to a second activation signal *ex vivo* in adipose macrophages from previously obese mice. Moreover, these data suggest that weight loss derived signals may act as priming stimuli to elicit changes in adipose macrophage metabolism and subsequent cytokine production that are consistent with innate immune memory.

### Weight cycling further increases inflammation in adipose macrophages

**Figure 5:**
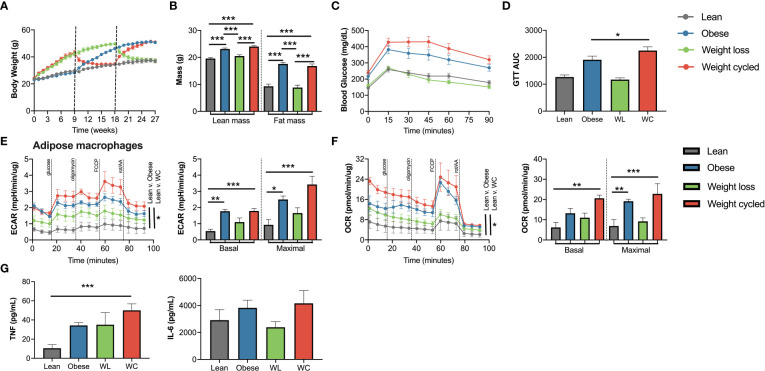
Weight cycling augments cell metabolism and basal cytokine production from adipose macrophages. Lean, obese, weight loss (WL) and weight cycled (WC) mice were generated over 27 weeks as in **Figure 1A**. **(A)** Body mass over time measured weekly with diet switch indicated by dashed lines. **(B)** Lean and fat mass measured by nuclear magnetic resonance. **(C)** Blood glucose during an intraperitoneal glucose tolerance test (1.5 g dextrose/kg lean mass) at 27 weeks and **(D)** area under the curve. **(E)** Extracellular acidification rate (ECAR) and **(F)** oxygen consumption rate (OCR) of epididymal adipose macrophages selected by 2-houradherence during modified mitochondrial stress test by Seahorse metabolic analyzer. **(G)** TNF and IL-6 production over 24 hours by ELISA in adipose macrophages selected by 2-houradherence and normalized by protein concentration. Data are means ± SEM of 10-12 mice for **(A–D)**, 5-14 mice for **(E–G)**, representative of 2 independent experiments. *p<0.05, **p< 0.01, ***p<0.001.

To determine if weight regain provides a sufficient secondary activation signal for innate memory *in vivo*, we utilized our previously published model of weight cycling as shown in **Figure 1A** (20, 21). As previously reported, obese vs. weight cycled mice and lean vs. weight loss mice had comparable body weight and body composition at the end of 27 weeks (**Figures 5A–B**). As we previously published, weight cycling also worsened glucose tolerance, to a greater degree than obesity alone, following an intraperitoneal glucose tolerance test as measured by area under the curve (**Figures 5C–D**). While there were no differences in epididymal adipose macrophage proportions in obese vs. weight cycled mice (**Figures S5A, B**), weight cycling increased basal glycolysis and oxidative phosphorylation in adipose macrophages compared to lean macrophages (**Figures 5E, F**). Maximal metabolism was significantly elevated compared to lean macrophages in both the obese and weight cycled groups, with weight cycled trending higher. Basal TNFα production from epididymal adipose macrophages was also elevated in the weight cycled condition compared with lean macrophages (**Figure 5G**). We also observed an increase in intracellular TNFα in weight cycled epididymal adipose macrophages by flow cytometry (**Figure S5C**). Moreover, subcutaneous adipose macrophages and liver macrophages have increased TNFα production following weight cycling (**Figures S6A, B**). Together, these data suggest that weight loss induces a form of innate immune memory that enhances TNFα production to weight regain as a secondary activation signal *in vivo*.

## Discussion

In the current study, we demonstrate that previous exposure to palmitic acid or conditioned media from obese adipose tissue in culture, as well as weight loss in mice, exacerbates the inflammatory response of adipose macrophages. We consider this a metabolic form of innate immune memory, similar to that previously shown in the context of diabetes and atherosclerosis (47–49). To our knowledge, this is the first study to directly link obesity, but more specifically weight loss, with innate immune memory.

While weight cycling worsens diabetes disease risk beyond stable long-term obesity, the mechanisms that link weight cycling and disease risk are not known. Using single-cell sequencing, we previously showed that after weight loss, adipose immune cells retain obesity-associated immunological changes like T cell exhaustion and macrophage lipid handing (21). Work by Singer, Hseuh, and Lumeng’s groups have shown that adipose macrophages are retained in the fat following weight loss and that they have greater inflammatory gene expression (50–52). In complementary work, oxidized LDL and Western diet increase LPS-responsiveness in monocytes and this is retained after switching to control diet for 4 weeks (49). In addition, 30% caloric restriction has been shown to protect mice against *Mycobacterium tuberculosis* infection (53). Our current work supports these studies by showing that weight loss does not reverse enhanced LPS-induced cytokine production by adipose macrophages. It should be noted that while we saw enhanced secondary activation in adipose macrophages from weight loss mice, we did not find that they secreted more basal cytokine by ELISA. These results partially differ from the reported increase in inflammatory gene expression by microarray data and in our single cell sequencing data; however, gene expression and protein do not always match, especially for cytokines which can be stored in their pro form prior to secretion. Moreover, our ELISA data came from cells mechanically and enzymatically isolated from the adipose tissue and cultured for 24 hours. Thus, future studies may take advantage of newer *in vivo* technologies to better understand basal cytokine production in adipose macrophages following weight loss. Importantly, these changes persist with long-term weight loss (12-24 weeks) (50, 52), suggesting they are long lasting and not simply in response to acute adipose tissue lipolysis or remodeling.

We propose this heightened inflammatory response is mediated by a metabolic form of innate immune memory. Initial reports of innate immune memory following exposure to β-glucan or BCG showed elevated glycolytic metabolism and inflammatory function (23, 54). We demonstrate similar changes in both cell culture and animal models, observing an increase in glycolysis and LPS-induced cytokine production. Priming with palmitic acid increases the response to many innate stimuli, including TLR2, TLR4, and dectin-1 agonists. Napier and colleagues have also shown that other models of palmitate treatment can augment macrophage inflammation in culture and induce an innate memory-like effect (55). Moreover, her group showed that dietary palmitate can augment the inflammatory response to endotoxemia and improve the clearance of *Candida albicans in vivo*. Together, our data emphasize the notion that palmitate induced-innate immune memory is not specific to one specific antigen, but primes macrophages for a more general heightened inflammatory response (28). In our study, we also observed an increase in maximal oxidative metabolism, which has been shown following monophosphoryl lipid-A priming, which is a TLR4 agonist like palmitic acid (56, 57).

Here we provide an exploratory study of the mechanisms involved in palmitate training. This model of innate immune memory is dependent on TLR4 activation as well as methyltransferase and metabolic activity. Similar to ox-LDL training, we found that general methyltransferase inhibition with MTA prevented the induction of immune memory (48). However, future work should specifically identify the epigenetic modifications required for palmitic acid memory. Recently, ATAC-sequencing analysis was published from lean and obese adipose tissue macrophages suggesting a role for the transcription factor ETV5 in adipose macrophage activation (58). ETV5 was significantly decreased in adipose macrophages from obese mice and in BMDMs treated with palmitate. Additionally, palmitate has been shown to increase JMJD3, a histone demethylase, in BMDMs (59). Together, these data support the premise that palmitate and obesity can induce epigenetic changes in macrophages and future work should examine how innate immune memory to palmitate may be different.

Importantly, we also show that weight loss induces this metabolic form of innate immune memory *in vivo*, which may exacerbate inflammation and diabetes risk upon weight regain. Adipose macrophages produce more TNFα after weight regain, which could be one plausible link. Macrophage cytokine production can directly promote adipocyte lipolysis and impair insulin signaling in obesity (19), which suggests a local mechanism by which weight cycling impairs glucose tolerance. However, recent experiments within our group suggest weight cycling does not worsen peripheral insulin resistance compared to obese animals, but rather impairs pancreatic insulin secretion (60). It is therefore plausible that secreted factors could reach other organs like the pancreas to promote dysfunction. Even more provocative, immune cells could possibly migrate from adipose tissue to other tissues. Alternatively, weight loss mobilizes lipid release from the adipose tissue (61), and it is possible that metabolic macrophage memory development could occur in tissues like the pancreas. While we did not assess pancreatic macrophages in this study, we show that liver macrophages have increased TNFα production following weight cycling. A third possibility is that innate immune memory may be initiated in the bone marrow prior to recruitment upon weight regain, and recruited cells may also infiltrate the pancreas. Interestingly, other models of innate immune memory occur in the bone marrow through IL-1β and IFN signaling (62); however, we cannot consistently measure cytokines in the plasma of our mice. Future work will directly interrogate these potential mechanisms to link enhanced macrophage inflammation, weight cycling, and worsened glucose tolerance.

There are a few noteworthy limitations to this study. First, only male mice were used in weight cycling studies in this report. While palmitic acid induced innate immune memory in both male and female BMDMs used for cell culture experiments, our published data indicate only a small change in glucose tolerance in female mice following weight cycling (21). There was only a very modest increase in iAUC for glucose tolerance compared with the obese mice (7833 v. 9994, p= 0.04). In the literature, it is well established that young female mice have very little weight gain and adipose expansion upon an initial exposure to high fat diet, and thus, the magnitude of initial priming may be important. We are currently testing new models to determine if aging, different time on diet, or gonadectomy affect weight cycling in female mice. Interestingly, the study by Napier and colleagues showed that both a ketogenic diet and palmitate oral gavage can induce innate immune memory and worsen LPS-induced sepsis in female mice (55). Coupled with the data that lipolysis mobilizes different lipid species in males and female mice (63) and the fact that female mice have lessened macrophage inflammation with weight gain and weight loss (51), these data provide support for our hypothesis that weight loss releases palmitate from male adipose tissue which may be [at least] one stimuli for innate immune memory in our model. Future work may further study the role of sex hormones or lipid species in the induction of innate immune memory with palmitic acid and weight cycling.

A second limitation is that it is not known whether metabolic memory is observed in human weight cycling or the extent to which humans would need to lose and gain weight. One final limitation is that our model relies on a switch from high fat to low fat diets. We have observed worsened glucose tolerance in response to pair feeding (weight loss on high fat diet) (60), and we expect other models of weight loss with lipid mobilization to prime adipose macrophages; however, we have not assessed macrophage activation following other models of weight loss like calorie restriction or bariatric surgery. Future experiments should work to further identify metabolic memory in female mice, humans, and following different types of weight loss, including weight loss following gastric bypass, drug therapy, exercise, or other diets.

Taken together, our data add to the growing body of literature which shows that while weight loss restores systemic glucose tolerance, it does not restore the adipose immune landscape. We also identify palmitic acid, adipose conditioned media, and weight loss as stimuli for innate immune memory development. This is physiologically important because weight regain worsens risk for diabetes and other diseases, and adipose macrophage metabolic memory may be one mechanistic link for this association. Future studies should investigate the causal role of metabolic memory and find potential therapeutic targets to alleviate weight cycling-accelerated disease.

## Data availability statement

The datasets presented in this study can be found in online repositories. The names of the repository/repositories and accession number(s) can be found in the article/**Supplementary Material**.

## Ethics statement

The animal study was reviewed and approved by Vanderbilt University Institutional Animal Care and Use Committee.

## Author contributions

HC conceptualized the study, obtained and analyzed the data, and drafted the manuscript. MC donated tissue from a few mouse cohorts and assisted with study design, sequencing data collection, and data interpretation. JP and LB assisted with culture model troubleshooting, data collection, and sequencing analysis. AH assisted with study design, provided funding, and is the guarantor for this work. All authors contributed to manuscript revisions. All authors read and approved the final manuscript.

## References

[1] Vidal J. Updated review on the benefits of weight loss. Int J Obes Relat Metab Disord (2002) 26 Suppl:4, S25–28. doi: 10.1038/sj.ijo.0802215 12457296

[2] Ryan DH Yockey SR. Weight loss and improvement in comorbidity: Differences at 5%, 10%, 15%, and over. Curr Obes Rep (2017) 6:187. doi: 10.1007/s13679-017-0262-y 28455679 PMC5497590

[3] de Leiva A. What are the benefits of moderate weight loss? Exp Clin Endocrinol Diabetes (1998) 106 Suppl:2, 10–13. doi: 10.1055/s-0029-1212030 9792475

[4] Wing RR Phelan S. Long-term weight loss maintenance. Am J Clin Nutr (2005) 82:222S–5S. doi: 10.1093/ajcn/82.1.222S 16002825

[5] Fildes A Charlton J Rudisill C Littlejohns P Prevost AT Gulliford MC. Probability of an obese person attaining normal body weight: Cohort study using electronic health records. Am J Public Health (2015) 105:e54–59. doi: 10.2105/AJPH.2015.302773 PMC4539812 26180980

[6] Crawford D Jeffery RW French SA. Can anyone successfully control their weight? findings of a three year community-based study of men and women. Int J Obes Relat Metab Disord (2000) 24:1107–10. doi: 10.1038/sj.ijo.0801374 11033978

[7] Stunkard A. The results of treatment for obesity: A review of the literature and report of a series. A.M.A. Arch Internal Med (1959) 103:79. doi: 10.1001/archinte.1959.00270010085011 13605305

[8] Bangalore S Fayyad R Laskey R DeMicco DA Messerli FH Waters DD. Body-weight fluctuations and outcomes in coronary disease. New Engl J Med (2017) 376:1332–40. doi: 10.1056/NEJMoa1606148 28379800

[9] Delahanty LM Pan Q Jablonski KA Aroda VR Watson KE Bray GA. Effects of weight loss, weight cycling, and weight loss maintenance on diabetes incidence and change in cardiometabolic traits in the diabetes prevention program. Diabetes Care (2014) 37:2738–45. doi: 10.2337/dc14-0018 PMC4170126 25024396

[10] Montani J-P Schutz Y Dulloo AG. Dieting and weight cycling as risk factors for cardiometabolic diseases: who is really at risk? Obes Rev (2015) 16 Suppl:1, 7–18. doi: 10.1111/obr.12251 25614199

[11] Rhee E-J Cho JH Kwon H Park SE Park C-Y Oh K-W. Increased risk of diabetes development in individuals with weight cycling over 4 years: The kangbuk Samsung health study. Diabetes Res Clin Pract (2018) 139:230–8. doi: 10.1016/j.diabres.2018.03.018 29574105

[12] Rzehak P Meisinger C Woelke G Brasche S Strube G Heinrich J. Weight change, weight cycling and mortality in the ERFORT Male cohort study. Eur J Epidemiol. (2007) 22:665–73. doi: 10.1007/s10654-007-9167-5 17676383

[13] Weisberg SP McCann D Desai M Rosenbaum M Leibel RL Ferrante AW. Obesity is associated with macrophage accumulation in adipose tissue. J Clin Invest (2003) 112:1796–808. doi: 10.1172/JCI200319246 PMC296995 14679176

[14] Russo L Lumeng CN. Properties and functions of adipose tissue macrophages in obesity. Immunology (2018) 155:407–17. doi: 10.1111/imm.13002 PMC6230999 30229891

[15] Ferrante AW. The immune cells in adipose tissue. Diabetes Obes Metab (2013) 15:34–8. doi: 10.1111/dom.12154 PMC3777665 24003919

[16] Jang JE Ko MS Yun J-Y Kim M-O Kim JH Park HS. Nitric oxide produced by macrophages inhibits adipocyte differentiation and promotes profibrogenic responses in preadipocytes to induce adipose tissue fibrosis. Diabetes (2016) 65:2516–28. doi: 10.2337/db15-1624 27246913

[17] Thomas D Apovian C. Macrophage functions in lean and obese adipose tissue. Metabolism (2017) 72:120–43. doi: 10.1016/j.metabol.2017.04.005 PMC5516622 28641779

[18] Bing C. Is interleukin-1β a culprit in macrophage-adipocyte crosstalk in obesity? Adipocyte (2015) 4:149. doi: 10.4161/21623945.2014.979661 26167419 PMC4496963

[19] Saltiel AR Olefsky JM. Inflammatory mechanisms linking obesity and metabolic disease. J Clin Invest (2017) 127:1–4. doi: 10.1172/JCI92035 28045402 PMC5199709

[20] Anderson EK Gutierrez DA Kennedy A Hasty AH. Weight cycling increases T-cell accumulation in adipose tissue and impairs systemic glucose tolerance. Diabetes (2013) 62:3180–8. doi: 10.2337/db12-1076 PMC3749340 23733197

[21] Cottam M Caslin H Winn N Hasty A. Multiomics reveals persistence of obesity-associated immune cell phenotypes in adipose tissue during weight loss and subsequent weight regain. Nat Commun (2022) vol. 13. doi: 10.1038/s41467-022-30646-4 PMC9135744 35618862

[22] Netea MG Quintin J van der Meer JWM. Trained immunity: A memory for innate host defense. Cell Host Microbe (2011) 9:355–61. doi: 10.1016/j.chom.2011.04.006 21575907

[23] Kleinnijenhuis J Quintin J Preijers F Joosten LAB Ifrim DC Saeed S. Bacille calmette-guérin induces NOD2-dependent nonspecific protection from reinfection Via epigenetic reprogramming of monocytes. Proc Natl Acad Sci U.S.A. (2012) 109:17537–42. doi: 10.1073/pnas.1202870109 PMC3491454 22988082

[24] Quintin J Saeed S Martens JHA Giamarellos-Bourboulis EJ Ifrim DC Logie C. Candida albicans infection affords protection against reinfection Via functional reprogramming of monocytes. Cell Host Microbe (2012) 12:223–32. doi: 10.1016/j.chom.2012.06.006 PMC3864037 22901542

[25] Rodriguez RM Suarez-Alvarez B Lopez-Larrea C. Therapeutic epigenetic reprogramming of trained immunity in myeloid cells. Trends Immunol (2019) 40:66–80. doi: 10.1016/j.it.2018.11.006 30595189

[26] Mulder WJM Ochando J Joosten LAB Fayad ZA Netea MG. Therapeutic targeting of trained immunity. Nat Rev Drug Discovery (2019) 18(7):553–66. doi: 10.1038/s41573-019-0025-4 PMC7069501 30967658

[27] van der Heijden CDCC Keating ST Groh L Joosten LAB Netea MG Riksen NP. Aldosterone induces trained immunity: the role of fatty acid synthesis. Cardiovasc Res (2020) 116:317–28. doi: 10.1093/cvr/cvz137 31119285

[28] Netea MG Domínguez-Andrés J Barreiro LB Chavakis T Divangahi M Fuchs E. Defining trained immunity and its role in health and disease. Nat Rev Immunol (2020) 20:375–88. doi: 10.1038/s41577-020-0285-6 PMC7186935 32132681

[29] Kashima DT Grueter BA. Toll-like receptor 4 deficiency alters nucleus accumbens synaptic physiology and drug reward behavior. Proc Natl Acad Sci U.S.A. (2017) 114:8865–70. doi: 10.1073/pnas.1705974114 PMC5565449 28760987

[30] Hao Y Hao S Andersen-Nissen E Mauck WM Zheng S Butler A. Integrated analysis of multimodal single-cell data. Cell (2021) 184:3573–3587.e29. doi: 10.1016/j.cell.2021.04.048 34062119 PMC8238499

[31] Ge SX Jung D Yao R. ShinyGO: a graphical gene-set enrichment tool for animals and plants. Bioinformatics (2020) 36:2628–9. doi: 10.1093/bioinformatics/btz931 PMC7178415 31882993

[32] Orr JS Kennedy AJ Hasty AH. Isolation of adipose tissue immune cells. JoVE (Journal Visualized Experiments) (2013) 75:e50707. doi: 10.3791/50707 PMC3718226 23728515

[33] Cao Y Dong Q Wang D Liu Y Zhang P Yu X. TIDB: a comprehensive database of trained immunity. Database (Oxford) (2021) 2021. doi: 10.1093/database/baab041 PMC8271126 34244719

[34] Kong L Moorlag SJCFM Lefkovith A Li B Matzaraki V van Emst L. Single-cell transcriptomic profiles reveal changes associated with BCG-induced trained immunity and protective effects in circulating monocytes. Cell Rep (2021) 37:110028. doi: 10.1016/j.celrep.2021.110028 34788625 PMC8728743

[35] Zhang B Moorlag SJCFM Domínguez-Andrés J Bulut Ö Kilic G Liu Z. Single-cell RNA sequencing reveals induction of distinct trained immunity programs in human monocytes. J Clin Invest (2022) 132:e147719. doi: 10.1172/JCI147719 35133977 PMC8970681

[36] Kar UK Joosten LAB. Training the trainable cells of the immune system and beyond. Nat Immunol (2020) 21:115–9. doi: 10.1038/s41590-019-0583-y 31925389

[37] Korbecki J Bajdak-Rusinek K. The effect of palmitic acid on inflammatory response in macrophages: an overview of molecular mechanisms. Inflamm Res (2019) 68:915–32. doi: 10.1007/s00011-019-01273-5 PMC6813288 31363792

[38] Coats BR Schoenfelt KQ Barbosa-Lorenzi VC Peris E Cui C Hoffman A. Metabolically activated adipose tissue macrophages perform detrimental and beneficial functions during diet-induced obesity. Cell Rep (2017) 20:3149–61. doi: 10.1016/j.celrep.2017.08.096 PMC5646237 28954231

[39] Kratz M Coats BR Hisert KB Hagman D Mutskov V Peris E. Metabolic dysfunction drives a mechanistically distinct pro-inflammatory phenotype in adipose tissue macrophages. Cell Metab (2014) 20:614–25. doi: 10.1016/j.cmet.2014.08.010 PMC4192131 25242226

[40] Seufert AL Hickman JW Traxler SK Peterson RM Waugh TA Lashley SJ. Dietary palmitic acid induces innate immune memory via ceramide production that enhances severity of acute septic shock and clearance of infection |. bioRxiv (2022). doi: 10.1101/2021.06.15.448579

[41] Wager CML Hole CR Campuzano A Castro-Lopez N Cai H Caballero Van Dyke MC. IFN-γ immune priming of macrophages in vivo induces prolonged STAT1 binding and protection against cryptococcus neoformans. PloS Pathog (2018) 14:e1007358. doi: 10.1371/journal.ppat.1007358 30304063 PMC6197699

[42] Edgar L Akbar N Braithwaite AT Krausgruber T Gallart-Ayala H Bailey J. Hyperglycemia induces trained immunity in macrophages and their precursors and promotes atherosclerosis. Circulation (2021) 144:961–82. doi: 10.1161/CIRCULATIONAHA.120.046464 PMC8448412 34255973

[43] Thiem K Stienstra R Riksen NP Keating ST. Trained immunity and diabetic vascular disease. Clin Sci (2019) 133:195–203. doi: 10.1042/CS20180905 30659160

[44] Sohrabi Y Sonntag GVH Braun LC Lagache SMM Liebmann M Klotz L. LXR activation induces a proinflammatory trained innate immunity-phenotype in human monocytes. Front Immunol (2020) 11. doi: 10.3389/fimmu.2020.00353 PMC7077358 32210962

[45] Boutens L Hooiveld GJ Dhingra S Cramer RA Netea MG Stienstra R. Unique metabolic activation of adipose tissue macrophages in obesity promotes inflammatory responses. Diabetologia (2018) 61:942–53. doi: 10.1007/s00125-017-4526-6 PMC6448980 29333574

[46] Serbulea V Upchurch CM Schappe MS Voigt P DeWeese DE Desai BN. Macrophage phenotype and bioenergetics are controlled by oxidized phospholipids identified in lean and obese adipose tissue. Proc Natl Acad Sci (2018) 115:E6254–63. doi: 10.1073/pnas.1800544115 PMC6142199 29891687

[47] Thiem K Keating ST Netea MG Riksen NP Tack CJ van Diepen J. Hyperglycemic memory of innate immune cells promotes in vitro proinflammatory responses of human monocytes and murine macrophages. J.I. (2021) 206:807–13. doi: 10.4049/jimmunol.1901348 33431659

[48] Bekkering S Quintin J Joosten LAB van der Meer JWM Netea MG Riksen NP. Oxidized low-density lipoprotein induces long-term proinflammatory cytokine production and foam cell formation via epigenetic reprogramming of monocytes. Arterioscler Thromb Vasc Biol (2014) 34:1731–8. doi: 10.1161/ATVBAHA.114.303887 24903093

[49] Christ A Günther P Lauterbach MAR Duewell P Biswas D Pelka K. Western Diet triggers NLRP3-dependent innate immune reprogramming. Cell (2018) 172:162–175.e14. doi: 10.1016/j.cell.2017.12.013 29328911 PMC6324559

[50] Zamarron BF Mergian TA Cho KW Martinez-Santibanez G Luan D Singer K. Macrophage proliferation sustains adipose tissue inflammation in formerly obese mice. Diabetes db160500 (2016) 66:392–406. doi: 10.2337/db16-0500 PMC5248991 28108608

[51] Griffin C Hutch CR Abrishami S Stelmak D Eter L Li Z. Inflammatory responses to dietary and surgical weight loss in male and female mice. Biol Sex Differ (2019) 10:16. doi: 10.1186/s13293-019-0229-7 30944030 PMC6446331

[52] Blaszczak AM Bernier M Wright VP Gebhardt G Anandani K Liu J. Obesogenic memory maintains adipose tissue inflammation and insulin resistance. Immunometabolism (2020) 2:e200023. doi: 10.20900/immunometab20200023 32774894 PMC7409818

[53] Palma C La Rocca C Gigantino V Aquino G Piccaro G Di Silvestre D. Caloric restriction promotes immunometabolic reprogramming leading to protection from tuberculosis. Cell Metab (2021) 33:300–318.e12. doi: 10.1016/j.cmet.2020.12.016 33421383

[54] Garcia-Valtanen P Guzman-Genuino RM Williams DL Hayball JD Diener KR. Evaluation of trained immunity by β-1, 3-glucan on murine monocytes in vitro and duration of response in vivo. Immunol Cell Biol (2017) 95:601–10. doi: 10.1038/icb.2017.13 PMC5550561 28228641

[55] Seufert AL Hickman JW Traxler SK Peterson RM Waugh TA Lashley SJ. Enriched dietary saturated fatty acids induce trained immunity. via ceramide production that enhances severity endotoxemia clearance infection. Elife (2022) 11:e76744. doi: 10.7554/eLife.76744 36264059 PMC9642993

[56] Fensterheim BA Young JD Luan L Kleinbard RR Stothers CL Patil NK. The TLR4 agonist monophosphoryl lipid a drives broad resistance to infection Via dynamic reprogramming of macrophage metabolism. J Immunol (2018) 200:3777–89. doi: 10.4049/jimmunol.1800085 PMC5964009 29686054

[57] Owen AM Fults JB Patil NK Hernandez A Bohannon JK. TLR agonists as mediators of trained immunity: Mechanistic insight and immunotherapeutic potential to combat infection. Front Immunol (2020) 11. doi: 10.3389/fimmu.2020.622614 PMC7930332 33679711

[58] Hu R-D Zhang W Li L Zuo Z-Q Ma M Ma J-F. Chromatin accessibility analysis identifies the transcription factor ETV5 as a suppressor of adipose tissue macrophage activation in obesity. Cell Death Dis (2021) 12:1023. doi: 10.1038/s41419-021-04308-0 34716308 PMC8556336

[59] Davis FM denDekker A Kimball A Joshi A Boniakowski A Kunkel S. Palmitate-TLR4 signaling regulates epigenetic modifications in macrophages and impairs diabetic wound healing. J Vasc Surg (2019) 70:e58–9. doi: 10.1016/j.jvs.2019.06.132

[60] Winn NC Cottam MA Bhanot M Caslin HL Garcia JN Arrojo E. Weight cycling impairs pancreatic insulin secretion but does not perturb whole-body insulin action in diet-induced obese mice. Diabetes db220161 (2022) 71:2313–30. doi: 10.2337/db22-0161 PMC9630085 35802127

[61] Kosteli A Sugaru E Haemmerle G Martin JF Lei J Zechner R. Weight loss and lipolysis promote a dynamic immune response in murine adipose tissue. J Clin Invest (2010) 120:3466–79. doi: 10.1172/JCI42845 PMC2947229 20877011

[62] Chen L Ozato K. Innate immune memory in hematopoietic Stem/Progenitor cells: Myeloid-biased differentiation and the role of interferon. Front Immunol (2021) 12:621333. doi: 10.3389/fimmu.2021.621333 33854500 PMC8039377

[63] Varghese M Griffin C McKernan K Eter L Lanzetta N Agarwal D. Sex differences in inflammatory responses to adipose tissue lipolysis in diet-induced obesity. Endocrinology (2019) 160:293–312. doi: 10.1210/en.2018-00797 30544158 PMC6330175

